# The Psychological Impact of Caring for a Parent With Cancer on Adult Children of South Asian Heritage: A Reflexive Thematic Analysis

**DOI:** 10.1002/pon.70557

**Published:** 2026-08-01

**Authors:** Carey Fagan, Emma Chapman, Gary Latchford

**Affiliations:** ^1^ Leeds Institute of Health Sciences University of Leeds Leeds UK

**Keywords:** cancer, caregiver, mental health, oncology, psychological impact, South Asian

## Abstract

**Objective:**

Caring for a parent with cancer is a common role taken on by adult children in the UK and has been linked to worsening mental health. Research has largely focused on White populations, however with an increase in people from minoritised backgrounds living in the UK, conducting research with populations that are representative of society is important. Therefore, this study aims to explore the psychological impact of caring for a parent with cancer on adult children of South Asian heritage.

**Method:**

Semi‐structured, face to face, in‐depth interviews were conducted with eight participants recruited from charity organisations in West Yorkshire. Reflexive thematic analysis was used to analyse the data.

**Results:**

The psychological impact described by participants was similar to research on other caregiving populations, but participants reported added challenges of managing community and familial expectations. In addition, participants reported difficult experiences with primary care potentially leading to later diagnosis. More positively, religion was mostly protective and helped participants cope.

**Conclusion:**

Elements of South Asian culture for example, expectations around caregiving and family values, adds to the psychological impact of caring for a parent with cancer. Untimely care from GPs and difficulties accessing support contributes to this. The benefits of implementing community psychology practices and service user involvement are considered, and suggestions made for improving accessibility to NHS psychology services.

## 1 Background

The rates of diagnosed cancers are increasing [1]. By 2050, it is estimated that 15% of the world's population will be over the age of 65 and over 19 million of these people are expected to develop cancer [2]. Advancements in diagnosing and treating cancer have increased survival rates. There are an estimated three million people living with cancer in the UK [3] and this is expected to rise [4]. To manage this, research needs to continue developing ways of screening and treating cancer but also harness ways of coping with an illness that will be experienced by 50% of the UK population at some point in their lives [5].

In recent years, there has been a shift from inpatient to outpatient care across the NHS [6] which has resulted in approximately 1 in 5 adults becoming caregivers [7]. When caring for a relative with cancer, family caregivers not only provide psychosocial support but are often required to help in the delivery of their relative's cancer care, which they are inadequately prepared for [2]. This has a considerable impact on their mental health and quality of life and can lead to caregivers experiencing equivalent or worse distress than the relative they are caring for [8]. It is estimated that cancer caregivers in the UK are 5 times more likely to have clinically significant anxiety and depression than the general population [9]. Despite the evidence of psychological distress their needs remain largely unmet and are often subsumed by the needs of the patient [10].

Roughly 80% of research on cancer caregiving has been on White populations [11]. However, culture has been found to have an impact on caregiver experience. Sociocultural stress and coping theory [12] suggests that psychological distress is not caused by the demands on caregivers but by the cultural appraisal of the demands, the norms in emotional expression and the social expectations. It is therefore argued that the psychological impact on caregivers from culturally diverse backgrounds may be underestimated because of reduced reporting of distress, even when the psychological impact is significant. Recent empirical work supports this position, concluding that carers from minority ethnic backgrounds often see their caregiving role through cultural and familial obligations [2, 13]. This can mean that people feel less able to express their emotions, have less autonomy and experience more symptoms of depression [2, 14]. In addition, carers from these groups are more likely to experience isolation, health, finance and employment difficulties resulting in increased psychological distress [15]. However, due to lower engagement with services because of ethnic inequalities [16] stigma around help‐seeking and differences in describing distress, the psychological impact of the role may be underreported and under detected [17].

The UK is home to many different ethnic groups that remain underrepresented in research [18]. Around 9.3% of the UK population have Asian heritage, which makes this group the largest minority ethnic group [19]. Within this group, the majority of people have South Asian heritage with the highest populations being Indian and Pakistani [20]. There is a mixed understanding of the needs of South Asian caregivers. Research has found that professionals may hold assumptions that British South Asian families prefer to keep the caring role within the family and are well supported by their community and faith [19]. This may lead to further inequalities in the support that caregivers can access and has been found to be inaccurate in dementia caregiving populations [19]. This research therefore aims to contribute to the field by providing a culturally accurate account of the psychological impact of caring for a parent with cancer on caregivers from a South Asian background living in the UK.

## 2 Method

### 2.1 Study Aims

This study aims to (1) explore the psychological impact of caring for a parent with cancer on adult children of South Asian heritage, (2) consider how NHS services can improve to support these caregivers.

### 2.2 Qualitative Approach and Research Paradigm

This study conducted a Reflexive Thematic Analysis [21]. A constructionist approach was taken, aligning with the view that there are multiple ways of interpreting people's realities based on individual world view and discourses within society [21]. This approach acknowledges the researcher's ‘active role’ in the development of the themes [22] and aligns with the study's relativist ontological position, that reality exists in our minds and is subjective [23].

### 2.3 Researcher Characteristics and Reflexivity

Power in an interview can be affected by class, education, profession, gender identity and ethnicity [24]. The researcher conducting the interviews disclosed their NHS affiliation. They also came from a White middle class background. The race‐of‐interviewer effect suggests that interviewee's will likely withhold information that may offend their interviewer of a different race [25]. In addition, there may have been an element of mistrust that could have prevented participants from being open about their experiences.

### 2.4 Expert by Experience (EBE) Involvement

To ensure this research empowered those who the research would affect [26] an expert by experience (EBE) was recruited. The EBE practitioner was from a Pakistani background and had lived in the UK her whole life. She cared for her mum with cancer until she died.

### 2.5 Procedure

Eligible participants were aged 18 or over, had a South Asian ethnic background, lived in the UK and had experience of caring for a parent with cancer. The term South Asian is used to describe ‘people whose familial or cultural backgrounds originate from India, Pakistan, Bangladesh, Nepal, Bhutan or Sri Lanka’ [27]. The relationship ‘parent’ included mother or father‐in‐law. Exclusion criteria included people who had cared for their parent outside of the UK. Eight participants were recruited in accordance with guidance from Braun and Clarke (2013; [28]). By the sixth interview, no new themes were identified and only minimal new codes, suggesting data saturation. Two more interviews were completed to confirm and enrich the thematic findings (see Table 1). Interviews lasted around 90 minutes. Data was collected between June and December 2023. A purposive sample was used to recruit people from South Asian community centres around West Yorkshire. This area was chosen due to its high population of South Asian people [29]. People were informed about the study via posters and visits to distribute information. Potential participants gave their consent to be contacted for an interview to a centre staff member, who informed the research team of their contact details. A topic guide based on relevant literature and informed by conversations with South Asian people in the community guided the interviews. Questions were open‐ended to facilitate discussion and capture the participant's experience.

**TABLE 1:** Data saturation table.

Interview	New codes identified	New themes identified	Notes
1	Substantial	Preliminary themes emerging	Broad range of codes. Initial patterns identified.
2	Moderate	Preliminary themes identified	Development of initial patterns using additional codes identified.
3	Minimal	Main themes identified	Development of initial themes and relationship to each other.
4	Minimal	No new themes—overarching theme identified	Developed final themes and relationship to each other.
5	Minimal	No new themes	Developed coherence among the themes under overarching theme.
6	Minimal	No new themes	Development of conceptual depth.
7	None	No new themes	Only confirming data identified suggesting data saturation.
8	None	No new themes	Only confirming data identified suggesting data saturation.

#### 2.5.1 Ethical Approval

This research project was approved by University of Leeds School of Medicine Research Ethics Committee (MREC—22‐073, 25/05/23).

### 2.6 Data Processing and Analysis

Interviews were recorded on an encrypted Dictaphone and then transcribed verbatim. All data was stored on the University of Leeds secure One Drive server. All paper data (consent forms, contact information and notes) were uploaded to the One Drive server and shredded. The server is managed by the University of Leeds Doctor of Clinical Psychology Course staff who delete any data stored after 3 years of completing the research. Interviews were analysed throughout data collection. During interviews, notes were made, highlighting recurring patterns and meaningful data. The six stages of thematic analysis (familiarisation, initial coding, generating themes, reviewing themes, defining themes and writing up) [21] were followed. A primarily inductive approach was taken to coding, meaning the data was ‘open‐coded’ to embody the experience described by the participants [30]. Elements of deductive analysis were also used to ensure the codes were relevant to the research question. To reduce ‘single researcher’ bias, each stage of the analysis was discussed with the research team. Codes and themes were analysed and adapted throughout, to ensure that they were reflective of the findings. Our expert by experience practitioner also reviewed the themes and made suggestions to improve cultural accuracy.

## 3 Results

**FIGURE 1:**
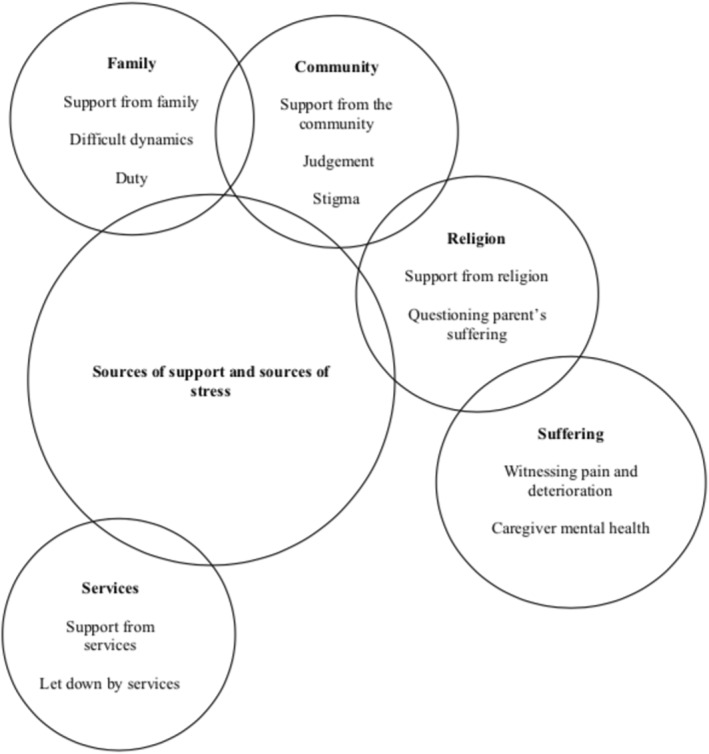
Thematic map of developed themes.

Eight participants were interviewed. See Table 2 for participant demographics. Despite all participants caring for a parent, the caregiving relationships varied in terms of intensity and duration. Some people were living with their parents and responsible for the majority of their care, whilst others were able to share the role within the family and live independently. Although it would be expected for this to reduce the psychological impact, it came with its own challenges of struggling to maintain different roles and other cultural expectations. For some caregivers, their parent's illness developed gradually over years but for others it was more of a rapid decline. The older generation of participant's spoke about differences in the understanding of cancer and how this impacted their distress at the time of caring. Thematic analysis resulted in one overarching theme titled ‘sources of support and sources of stress’, overlapping with four themes: ‘services’, ‘family’, ‘community’, and ‘religion’. The fifth theme (‘suffering’) did not overlap with the overarching theme. See Figure 1 for the thematic map.

**TABLE 2:** Participant demographics (*n* = 8).

Gender	Age	Employment status	Relationship status	Country of birth	Heritage	Years living in the UK	Religious faith	Parent's cancer type	Outcome of parent's illness
Female (7)	35–44 (3)	Full‐time employment (3)	Married (4)	Pakistan (3)	Pakistani (5)	Whole life (3)	Muslim (7)	Brain tumour (1)	Survivorship (1)
Male (1)	45–54 (2)	Part‐time employment (2)	Divorced (2)	UK (3)	Bangladeshi (2)	50+ years (1)	Sikh (1)	Prostate cancer (1)	Died (7)
	55–64 (2)	Unemployed (2)	Single (2)	Bangladesh (2)	Indian (1)	46–50 years (1)		Bone marrow cancer (1)	
	65–74 (1)	Retired (1)				41–45 years (1)		Peritoneal cancer (1)	
						36–40 years (1)		Liver cancer (2)	
						6–10 years (1)		Lung cancer (1)	
								Pancreatic cancer (1)	

### 3.1 Services

#### 3.1.1 Support From Services

Palliative care was spoken about positively alongside hospitals and third sector organisations. Respectful and compassionate care helped caregivers feel supported and reduced caregiver distress: ‘The palliative team, they were amazing. The hospice couldn't have left her in better care for the nights when she needed it’. Secondary care services (hospice care and NHS hospitals) were viewed, by some, as being culturally aware: ‘The hospital they'd say (Name) would you want the Indian menu…and I think if she wanted to go pray, she could, and they said if you want to bring your own priest to the hospital you can’. However, many participants did not discuss cultural competence. Some participants reported that they did not feel they needed the services to be culturally aware and presented as indifferent about the quality of care from that aspect.

#### 3.1.2 | Let Down by Services

Despite the impact the caregiving role had on participants mental health, there was minimal mention of psychological therapy services. Some people did not know how to access support whereas others chose not to. Of participants that had an intervention, one received counselling and found it helpful, and another received Cognitive Behaviour Therapy (CBT) which she states did not work for her ‘I did a couple [of CBT sessions]. It didn't really do it for me. I think more my religion is helpful, I think the CBT was good in that way where I was like having this conversation or like just talking to someone, but I couldn't figure it out’. Most participants, including those who had praised some services, spoke about ways services had let them down. Seven out of eight participants spoke about primary care services not diagnosing their relative early enough and felt as if they were not listened to: ‘He'd been to the doctors loads of times complaining of stomach pains and urine infections but nobody took us seriously….Until one day he was so ill they did some tests and biopsy, and they came back as (pause) they couldn't do anything for the cancer it was in its final fourth stage’.

### 3.2 Family

#### 3.2.1 Support From Family

Most participants said family support had a positive impact on their psychological wellbeing. This was due to the caregiving role being shared; financial and emotional support; and respite from having family around their home: ‘Sometimes my family members, they came to visit him. We also at that time we need support from other people! We need mentally … physically, everybody do it’. People spoke about how it brought them together and how they connected with each other because they were going through the same thing: ‘My sisters they kept me going, cos I think if my sisters weren't there, it would have been rock bottom. We supported each other and the extended family’.

#### 3.2.2 Difficult Dynamics

During times of stress, people reported difficult dynamics with family members including parents, sibling or with their partner and own children: ‘I have my own way of doing things. So, there's a lot of … clashes between me and dad. How to do things so that he's always second guessing … what I should be doing’. These dynamics caused notable distress to caregivers and were difficult to manage on top of their other responsibilities. Caregivers felt as if they were being pulled in different directions when trying to maintain family functioning. Female participants juggled caregiving alongside their role as mother or wife: ‘Well for me it was challenging, trying to keep my home life going and then caring for my dad and I felt like I was being pulled by both directions and then culturally, my husband and his family were saying it's not your responsibility it should be the son and that was making me feel even worse’. Cultural expectation that a parent should be cared for by the son, negatively impacted several participants.

#### 3.2.3 Duty

A strong sense of duty and cultural expectations were widely reported: ‘Culturally, I felt like, I had to do things according to this, in order to breathe or have this time for myself…But ultimately, I just, you know, as a daughter it naturally comes where everything else can be secondary’. People described pressure around their caregiving role but their duty to care did not necessarily worsen the psychological impact, despite having less autonomy. In some cases, it seemed to ease the burden as people spoke about it as if it was part of their identity: ‘It's about giving them comfort, making aware of the dignity, making sure they are respected. To be a carer for her was a blessing’. The positive notion of the caregiving role being a blessing was expressed by others, and this seemed to have a powerful impact on mental wellbeing.

### 3.3 Community

#### 3.3.1 Judgement

Along with duty, participants spoke about feeling judged by their community. Participants felt criticised for the way they cared for their parent which elicited feelings of anxiety and overwhelm: ‘But it does impact you because you don't need the extra pressure, you don't need the extra criticism, how are you caring for them, are you doing enough? Who's looking after them? It's none of your business how we care’. Community opinion was often described as insensitive and participants spoke about the impact it had on them: ‘Our own communities can be our own enemies like you just judge and stereotype and it's just idle gossip, but you don't look at what the person's feeling’. In addition, there was mention of communities being insensitive towards the ill parent: ‘They're not being sympathetic or caring towards him, they're like finger pointing or making…not fun of you but just making the situation worse than it is’. As would be anticipated this worsened the psychological impact on both caregivers and their parents.

#### 3.3.2 Stigma

There was mention of stigma from communities around mental health and cancer. People spoke about mental health not being understood. However, there were clear differences between generations and a theme of things changing: ‘They don't understand the concept of mental health…some don't but others did’. Some of the older participants who cared for their parent earlier in their lives shared that their families did not want people from the community to know that their parent was ill because there was stigma around cancer being a terminal illness: ‘It's a very different, open conversation now. Whereas ours wasn't when I was caring … it was just really weird’. Participants spoke about younger generations having an improved understanding of cancer, which eased the psychological impact of a diagnosis on patient and caregivers: ‘Cancer was a word that's not supposed to be spoken. But now it isn't, now people have opened up, they do tell you’.

#### 3.3.3 Support From the Community

Despite the negative impact community involvement could have, participants also spoke about support that they received from their community and how this helped their parent's mental health: ‘It was irritating, it was tyring, but it was nice that they were there cos sometimes when people were sat in here you could go and have that ten‐minute nap, so that support from the community, as hard as it was, it was a support to some extent as well’. Communities help by keeping their parent company or bringing food. However, there were still threads of a negative impact as caregivers reported having to host people which was an unwanted additional task.

### 3.4 Religion

#### 3.4.1 Support From Religion

Many participants spoke about using religion to help them cope. Carers increased the amount they prayed: ‘I was praying a lot more than what I'd normally do. My prayers and everything became consistent. I think that's helped me to cope’. In addition, people spoke about believing that praying could help their unwell parent, which seemed to soothe feelings of helplessness: ‘In our religion we believe like maybe someone's prayer for my Dad … might help’. Participant's faith mostly had a positive impact on mental health and there was a strong sense of acceptance throughout: ‘I trust that it’s God up there and I know there's a reason why we're here. So, these are all the ideologies and beliefs that I carry, and I strongly do believe that this is my journey, and this is my test’. People placed a lot of power in their God and believed that things were always going to be the way that they were. Believing in ‘God's will’ helped people cope with the death of their parent: ‘I kind of believe like if it's meant to be, it's meant to be. That's what's written for you…nothing is in your control, so the outcome doesn't matter’. Participants did not feel responsible for their parent's suffering or dying experience. Any control over the situation was viewed in the hands of God.

#### 3.4.2 Questioning Parent's Suffering

Despite participant's believing it was God's will for their parents to die, there was turmoil over the way their parent's suffered: ‘She'd [her mum] say why did God punish me and why won't he just take me why is he making me suffer… she did really good and then why did he make her suffer for 3 years’. This had an impact on caregiver distress and people spoke about the emotional toll of this. For the Sikh participant this questioning was stronger because of their religious belief in karma. This idea that it could have been their parent's fault that they suffered was very painful, and it seemed to create anguish when trying to make sense of the experience.

### 3.5 Suffering

#### 3.5.1 Witnessing Pain and Deterioration

People spoke about the physical deterioration of their parents. As would be expected, witnessing this, especially when a parent was in pain, caused significant distress: ‘For me seeing it.. I used to feel really tight in my chest, it felt like it's been you know cut open every time and I read somewhere that they said daughters feel the pain of their fathers’. Many participants described their suffering in this way, as if they were physically holding the pain in themselves, highlighting a strong connection between child and parent. Sadly, the majority of participants' parents died: ‘It's too hard to watch your… loved ones, to like in front of your eyes, like a dying person’. Witnessing death had a considerable psychological impact. There was a sense of feeling out of control and helpless in those moments when there was nothing they could do but witness the loss of their loved one.

#### 3.5.2 Caregiver's Mental Health

Participants spoke about the role making them mentally unwell. People seemed to identify most with having anxiety and/or depression: ‘It's depression mainly. But I do get … anxiety. Erm you get kind of pangs of it, but I can recognise that it’s happening now. But depression is … erm, I'm up and down all the time’. There were other participants who described not being able to recognise their symptoms of mental ill health. This worsened the impact it had on them because they were not sure what they were experiencing, which in some cases prevented them seeking help. Participants spoke about the lasting impact losing their parent had on them: ‘It's like a part of you is missing when you lose someone, especially a figure like your mother who was meant to be strong, who was meant to be your world or meant to be this protector, now we're protecting’. Participants spoke about reversal of parent and child roles. They described their parents as strong leaders of their family that protected them growing up. The experience of seeing this person deteriorate and die was particularly challenging. There was a sense of disbelief as to how their parent could change so significantly from the illness, and even that their parent could become unwell at all.

## 4 Discussion

The psychological impact of caring for a parent with cancer on adult children of South Asian heritage is unsurprisingly complex. The findings highlight ways in which aspects of South Asian culture may be protective of caregiver's mental health. All participants had a strong faith that helped them cope. This is consistent with recent research findings on caregiving populations in India [31, 32] and highlights the need for professionals and services to recognise the intersection between religion, culture and caregiving rather than seeing them as separate influences. The emotional impact of witnessing a parent with cancer deteriorate and die, had a significant impact on mental health, suggesting that the psychological burden of caregiving may continue beyond the role itself. This finding aligns with broader cancer caregiving literature, which highlights how caregivers often experience anticipatory grief whilst watching their relative's physical decline and significant bereavement‐related distress afterwards [33].

Other cultural norms had a mixed impact on caregiver's mental health. Caregivers reported the challenges in managing negative and judgemental input from their community and families, as well as the stress caused by juggling multiple roles, responsibilities and relationships. However, in contrast to research on other caregiving populations [2, 14] their sense of cultural duty was generally reported to reduce distress. From a sociocultural stress and coping theory [12] perspective, this study therefore supports that cultural appraisals and social expectations have a significant impact on caregiver experience.

However, while a sense of duty may have reduced distress, it may have also contributed to participants not accessing mental health services. In addition, participants spoke about not knowing how to access support and not receiving culturally appropriate treatment when they did. These findings therefore not only reinforce theory around the impact of culture on caregiver experience, but they also challenge assumptions that some care professionals may have about South Asian culture and its role in cancer caregiver's mental health (that family, religion and the community are solely protective) when this may not be the case. In addition, the findings document the extreme distress people experience, and the unmet need for equitable professional support. These findings have several important clinical implications.

### 4.1 Implications for Practice

The high levels of distress reported, alongside low service uptake, suggest that reliance on self‐referral may be insufficient, and that routine screening of caregiver distress should be embedded within oncology services. Given the influence of cultural factors, there is a need for culturally adapted interventions that account for family roles, stigma, and culturally specific understandings of distress. Improving access to support at service‐level is critical; this may involve the use of community‐based outreach, language‐concordant practitioners, and alternative models of delivery that enhance engagement among South Asian caregivers. To facilitate these improvements, service user involvement should be used in oncology and psychology services. Research has shown how this can be successfully implemented to develop culturally competent services [34]. The findings also support a shift towards family‐centred care, where caregiver needs are assessed alongside those of patients. Finally, training for healthcare professionals in cultural competence is needed to improve identification of distress and effective treatment.

### 4.2 Study Limitations

This study used a small sample, so generalisability of the themes is limited. The term South Asian encapsulates a group of people from different cultural backgrounds and pooling people into this group risks ignoring those differences. Having participants from different religious backgrounds for example, Sikh and Muslim, created some diversity in results. This was managed by reporting individual experience where meaningful. Similarly, the sample only included one man, however no clear differences were found in his reported experience. The research team were all White which may have influenced interpretation of results. There may have been topics, such as finding services to be culturally insensitive, that participants may not have been comfortable to share.

### 4.3 Future Research

Future research should build on these findings in several ways. Longitudinal studies are needed to examine how caregiver distress evolves across the cancer trajectory and to identify critical timepoints for intervention. Qualitative research exploring barriers to help‐seeking, particularly among those who do not access services, would provide important insight into the low uptake reported. A peer‐interview method may be helpful so that participants feel able to be open up about these experiences. There is also a need to develop and evaluate culturally adapted psychological interventions tailored to South Asian caregivers, alongside studies testing strategies to improve engagement with services. Finally, given the central role of family, future work should also explore family‐based or dyadic intervention models.

## 5 Conclusion

Adult South Asian caregivers suffer significantly from the psychological impact of caring for a parent with cancer. The emotional suffering reported in this study mirrored research on other caregiving populations but in addition, caregivers reported added challenges of managing community and familial expectations. Participants cultural appraisal of their role had an impact on levels of distress and expression of this. More positively, religion was found to mostly be protective and helped participants to cope. The benefits of incorporating these coping mechanisms in any psychological intervention of caregiver distress is important. In addition, adapting service delivery and improving accessibility is needed to ensure that South Asian caregivers can access appropriate support for their mental health.

## Author Contributions

This is an in‐depth study conducted in the UK with underrepresented participants. It draws on sociocultural stress and coping theory to report a culturally accurate account of South Asian caregiver's experiences.

## Funding

The authors have nothing to report.

## Conflicts of Interest

The authors declare no conflicts of interest.

## Data Availability

The data that support the findings of this study are available on request from the corresponding author. The data are not publicly available due to privacy or ethical restrictions.

## References

[1] NHS England , NHS England» NHS Diagnoses Thousands More Cancers as Cases Rise by 5% (England.nhs.uk, 2024), https://www.england.nhs.uk/2024/10/nhs‐diagnoses‐thousands‐more‐cancers‐as‐cases‐rise‐by‐5/.

[2] V. Sun , M. Puts , K. Haase , et al., “The Role of Family Caregivers in the Care of Older Adults With Cancer,” Seminars in Oncology Nursing 37, no. 6 (December 2021): 151232: [Cited 2025 April 15], https://www.sciencedirect.com/science/article/abs/pii/S0749208121001479. 34753644 10.1016/j.soncn.2021.151232

[3] L. Warrington , K. Absolom , P. Baxter , et al., “Quality of Life, Healthcare Usage and Finances of UK Cancer Survivors Five Years Post‐Diagnosis: A Matched Controlled Study,” Journal of Cancer Survivorship (December 2024): [Cited 2025 November 22], https://pubmed.ncbi.nlm.nih.gov/39627376/. 10.1007/s11764-024-01708-x PMC13144260 39627376

[4] A. Aggarwal , A. Choudhury , N. Fearnhead , et al., “The Future of Cancer Care in the UK—Time for a Radical and Sustainable National Cancer Plan,” Lancet Oncology 25, no. 1 (January 2024): e6–e17: [Cited 2025 November 22], https://pubmed.ncbi.nlm.nih.gov/37977167/. 37977167 10.1016/S1470-2045(23)00511-9

[5] C. Vrinten , A. Gallagher , J. Waller , and L. A. V. Marlow , “Cancer Stigma and Cancer Screening Attendance: A Population Based Survey in England,” BMC Cancer 19, no. 1 (June 2019): 566: [Cited 2025 November 22], https://bmccancer.biomedcentral.com/articles/10.1186/s12885‐019‐5787‐x#citeas. 31185949 10.1186/s12885-019-5787-x PMC6561035

[6] L. M. Pettigrew , J. Beech , H. Alderwick , and J. Smith , “From Hospital to Community? The Good, the Bad, and the Ugly of the 10‐Year Plan to Move Care to the Community,”British Journal of General Practice 75, no. 759 (September 2025): 440–442: [Cited 2025 November 22], 10.3399/bjgp.2025.0567. PMC12754645 40998703

[7] Carers Week 2022 Report (2022): [Cited 2025 July 10], https://www.carersweek.org/media/qf0p5u4t/carers‐week‐2022‐make‐caring‐visible‐valued‐and‐supported‐report_final.pdf.

[8] E. Coyne , N. Heynsbergh , and K. B. Dieperink , “Acknowledging Cancer as a Family Disease: A Systematic Review of Family Care in the Cancer Setting,” European Journal of Oncology Nursing 49 (December 2020): 101841: [Cited 2025 April 20], https://www.sciencedirect.com/science/article/abs/pii/S1462388920301216. 33130424 10.1016/j.ejon.2020.101841

[9] E. Redican , R. Meade , C. Harrison , et al., “The Prevalence, Characteristics, and Psychological Wellbeing of Unpaid Carers in the United Kingdom,” Social Psychiatry and Psychiatric Epidemiology 60, no. 4 (August 2024): 869–879: [Cited 2025 November 21]; 10, https://link.springer.com/content/pdf/10.1007/s00127‐024‐02745‐8.pdf. 39126515 10.1007/s00127-024-02745-8 PMC12031964

[10] A. Williams and M. Bakitas , “Cancer Family Caregivers: A New Direction for Interventions,” Journal of Palliative Medicine 15, no. 7 (July 2012): 775–783: [Cited 2025 April 13], https://www.ncbi.nlm.nih.gov/pubmed/22612407. 22612407 10.1089/jpm.2012.0046 PMC3387766

[11] K. Schwartz , J. Beebe‐Dimmer , T. A. Hastert , et al., “Caregiving Burden Among Informal Caregivers of African American Cancer Survivors,” Journal of Cancer Survivorship 15, no. 4 (August 2021): 630–640: [Cited 2025 April 13], https://pubmed.ncbi.nlm.nih.gov/33067774/. 33067774 10.1007/s11764-020-00956-x PMC8052386

[12] B. G. Knight and P. Sayegh , “Cultural Values and Caregiving: The Updated Sociocultural Stress and Coping Model,” Journal of Gerontology: Psychological Science 65B, no. 1 (January 2010): 5–13: [Cited 2026 May 16], 10.1093/geronb/gbp096. 19934166

[13] C. Segrin , T. Badger , and A. Sikorskii , “Psychological Distress and Social Support Availability in Different Family Caregivers of Latinas With Breast Cancer,” Journal of Transcultural Nursing 31, no. 3 (March 2021): 269–278: [Cited 2026 May 16], https://pubmed.ncbi.nlm.nih.gov/31876235/. 10.1177/1043659619896824 31876235

[14] V. Lawrence , J. Murray , K. Samsi , and S. Banerjee , “‘The Mind May Go, But the Heart Knows’: Emotional Care by Ethnic Minority Carers of People Living With Dementia,” Social Science & Medicine 287 (August 2021): 114339: [Cited 2026 May 16], https://pubmed.ncbi.nlm.nih.gov/34365072/. 34365072 10.1016/j.socscimed.2021.114294

[15] M. Stephanou , “Caregiver Burden: Support Needed for Those Who Support Others and the National Health Service,” Patient Experience Journal 10, no. 2 (August 2023): 23–33: [Cited 2025 April 04], https://pxjournal.org/cgi/viewcontent.cgi?article=1796&context=journal.

[16] N. Bansal , S. Karlsen , S. P. Sashidharan , R. Cohen , C. A. Chew‐Graham , and A. Malpass , “Understanding Ethnic Inequalities in Mental Healthcare in the UK: A Meta‐Ethnography,” PLoS Medicine 19, no. 12 (October 2022): e1004139: [Cited 2025 May 09], https://pubmed.ncbi.nlm.nih.gov/36512523/. 36512523 10.1371/journal.pmed.1004139 PMC9746991

[17] P. Behera , N. Gill , H. Sultan , G. A. W. Heckman , and J. P. Hirdes , “Symptoms of Mood Disturbance and Depression Diagnosis Among South Asian Home Care Clients in Ontario, Canada: Evidence of Under‐Detection of Mental Health Needs,” International Journal of Geriatric Psychiatry 41, no. 3 (March 2026): e70201: [Cited 2026 June 24], 10.1002/gps.70201. 41841339 PMC12993800

[18] NHS England . NHS England» Not ‘Hard to Reach’—Increasing Diversity in Research Participation. (England.nhs.uk, 2023): [Cited 2025 June 22], https://www.england.nhs.uk/blog/not‐hard‐to‐reach‐.

[19] ONS . Ethnic Group, England and Wales—Office for National Statistics (Office for National Statistics, 2022) [Cited 2025 November 22], https://www.ons.gov.uk/peoplepopulationandcommunity/culturalidentity/ethnicity/bulletins/ethnicgroupenglandandwales/census2021.

[20] T. James , N. Mukadam , A. Sommerlad , S. Barrera‐Caballero , and G. Livingston , “Equity in Care and Support Provision for People Affected by Dementia: Experiences of People From UK South Asian and White British Backgrounds,” International Psychogeriatrics 36, no. 7 (July 2024): 564–573: [Cited 2025 July 10], https://pubmed.ncbi.nlm.nih.gov/36803586/. 36803586 10.1017/S1041610223000121

[21] V. Braun and V. Clarke , “Using Thematic Analysis in Psychology,” Qualitative Research in Psychology 3, no. 2 (2006): 77–101, 10.1191/1478088706qp063oa.

[22] V. Braun and V. Clarke , “Can I Use TA? should I Use TA? Should I *Not* Use TA? Comparing Reflexive Thematic Analysis and Other Pattern‐Based Qualitative Analytic Approaches,” Counselling and Psychotherapy Research 21, no. 1 (October 2020): 37–47: [Cited 2025 April 22], 10.1002/capr.12360.

[23] M. J. D. Levers , “Philosophical Paradigms, Grounded Theory, and Perspectives on Emergence,” Sage Open 3, no. 4 (December 2013): 1–6: [Cited May 01], 10.1177/2158244013517243.

[24] F. Anyan , “The Influence of Power Shifts in Data Collection and Analysis Stages: A Focus on Qualitative Research Interview,” Qualitative Report 18, no. 18 (January 2013): [Cited 2025 June 12], 10.46743/2160-3715/2013.1525.

[25] A. M. Enders and J. R. Thornton , “Biased Interviewer Assessments of Respondent Knowledge Based on Perceptions of Skin Tone,” Journal of Race Ethnicity and Politics 7, no. 3 (July 2022): 572–588: [Cited 2025 November 20], https://www.cambridge.org/core/journals/journal‐of‐race‐ethnicity‐and‐politics/article/biased‐interviewer‐assessments‐of‐respondent‐knowledge‐based‐on‐perceptions‐of‐skin‐tone/386AEE3B2C73508BCF689AABC0C6BCE0.

[26] J. R. Beames , K. Kikas , M. O’Gradey‐Lee , et al., “A New Normal: Integrating Lived Experience Into Scientific Data Syntheses,” Frontiers in Psychiatry 12 (October 2021): 763005: [Cited 2025 April 04], https://pmc.ncbi.nlm.nih.gov/articles/PMC8585932/. 34777064 10.3389/fpsyt.2021.763005 PMC8585932

[27] R. Prajapati and H. Liebling , “Accessing Mental Health Services: A Systematic Review and Meta‐Ethnography of the Experiences of South Asian Service Users in the UK,” Journal of Racial and Ethnic Health Disparities 9, no. 2 (April 2022): 598–619: [Cited 2025 April 29], https://pubmed.ncbi.nlm.nih.gov/33686621/. 33686621 10.1007/s40615-021-00993-x PMC8897382

[28] V. Braun and V. Clarke , Successful Qualitative Research a Practical Guide for Beginners (Sage, 2013).

[29] Census 2021. (Westyorks‐ca.gov.uk, 2021): [cited 2025 Dec 1], https://www.westyorks‐ca.gov.uk/data/census‐2021/.

[30] D. Byrne , “A Worked Example of Braun and Clarke’s Approach to Reflexive Thematic Analysis,”Quality and Quantity 56, no. 3 (June 2021): 1391–1412: [Cited 2025 July 10], 10.1007/s11135-021-01182-y.

[31] R. R. Angelin , S. Saravanan , F. C. James , and R. Samuel , “Experience of Religiosity in Caregiving for Persons With Serious Mental Illness: A Qualitative Study Using Interpretative Phenomenological Analysis From India,” BMJ Open 15, no. 3 (March 2025): e090838: [Cited 2026 July 10], 10.1136/bmjopen-2024-090838. PMC11907034 40081977

[32] W. Saba , M. Faran , J. Sardar , and S. Ashraf , “Caregivers” Burden and Mental Health of the Caregivers of β‐Major Thalassemia Patients: Mediating Role of Religious Coping,” Journal of Health and Rehabilitation Research 4, no. 2 (May 2024): 471–476: [Cited 2026 July 10], 10.61919/jhrr.v4i2.831.

[33] K. Svop , K. B. Dieperink , T. Livingston , and J. Marcussen , “Families Experience of Anticipatory Grief in Home‐Based Palliative Cancer Care and Their Support Needs: A Qualitative Study,” European Journal of Oncology Nursing 76 (March 2025): 102880: [Cited 2026 June 24], 10.1016/j.ejon.2025.102880. 40187033

[34] M. A. Tanay , J. Armes , C. Oakley , et al., “Co‐Designing a Cancer Care Intervention: Reflections of Participants and a Doctoral Researcher on Roles and Contributions,” Research Involvement and Engagement 8, no. 1 (August 2022): [Cited 2025 May 15], 10.1186/s40900-022-00373-7. PMC9343815 35918715

